# A cross sectional study examining social desirability bias in caregiver reporting of children’s oral health behaviors

**DOI:** 10.1186/1472-6831-13-24

**Published:** 2013-06-01

**Authors:** Lauren A Sanzone, Jessica Y Lee, Kimon Divaris, Darren A DeWalt, A Diane Baker, William F Vann Jr

**Affiliations:** 1 Department of Pediatric Dentistry, School of Dentistry, University of North Carolina, 228 Brauer Hall, Chapel Hill, NC, USA; 2 Division of General Internal Medicine, School of Medicine, University of North Carolina, 5041 Old Clinic Building, CB#7110 Chapel Hill, NC, USA

**Keywords:** Caregivers children, Oral health, Oral hygiene, Health literacy, Oral health literacy, Oral health behaviors, REALD-30, Social desirability bias

## Abstract

**Background:**

Our previous research (*Pediatrics* 2010:126) found a strong association between caregiver oral health literacy (OHL) and children’s oral health status; however, we found a weak association with oral health behaviors (OHBs). We hypothesize that this may be due to social desirability bias (SDB). Our objectives were to compare caregivers’ responses to traditional OHB items and newer SDB-modulating items, and to examine the association of caregiver literacy with OHBs.

**Methods:**

We performed a cross-sectional study of 102 caregiver-child dyads, collecting data for OHBs using both traditional and new SDB-modulating items. We measured OHL using REALD-30, a validated word recognition test. We relied upon percent agreement and Cohen’s *kappa* (k) to quantify the concordance in caregivers’ responses and multivariate log-binomial regression to estimate the impact of OHL on OHBs.

**Results:**

Caregivers’ mean REALD-30 score was 20.7 (SD = 6.0), range 1-30. We found an association between OHL and 4 of 8 OHBs examined. A subset of behavior questions compared traditional *versus* SDB-modulating items: history of bottle-feeding: agreement = 95%, k = 0.83 (95% CL:0.68,0.99); daily tooth brushing: agreement = 78%, k = 0.25 (95% CL:0.04,0.46); fluoridated toothpaste use: agreement = 88%, k = 0.67 (95% CL:0.49,0.85). After controlling for caregivers’ race, marital status and study site, higher literacy scores remained associated with a decreased prevalence of parental report of “decided not brush the child’s teeth because it would be frustrating”.

**Conclusions:**

Agreement between responses was high for 2 of 3 behavior items. Item 3 (tooth brushing frequency) revealed discordance, likely due to SDB. Use of the SDB-modulating items appears to yield a better estimate of OHB.

## Background

### Health literacy

Health literacy has been defined as “the capacity to obtain, process, and understand basic health information and services needed to make appropriate health decisions” [1]. The ability to interact effectively with our increasingly complex health care system requires a set of skills that is distinct from general literacy skills. These include writing, listening, oral communication, numeracy, and organization. Oral health literacy [1] is an extension of the concept into the oral health domain, relating to an individual’s ability to appropriately manage their oral health care needs.

The health literacy knowledge base has expanded in recent years, with enormous implications for health care delivery and intervention design. One of the key conclusions from this research has been that interventions are unlikely to result in significant change in individuals’ health literacy; however, improvements in patient communication and resultant health knowledge can be realized through enhanced written and multimedia communication, improved provider communication skills, and accommodations in systems of care [2,3].

### Health literacy and child health outcomes

It is also known that caregivers’ literacy impacts the health of their children. Low health literacy in adult caregivers can affect children’s health because children are dependent on their caregivers for access to health care and self-management support [4]. At home, young children need caregivers to perform preventive health behaviors such as toothbrushing and medication administration; older children may be able to perform these behaviors on their own, but still need supervision and encouragement. Additionally, maternal literacy skills have been shown to correlate highly with behaviors that promote health in infants and children, including avoidance of smoking, breastfeeding initiation, and compliance with immunization and preventive care schedules [5,6].

When compared to parents with higher literacy, those with lower literacy demonstrated less health knowledge, and behaviors that were less beneficial to their children’s health. Accordingly, children of caretakers with low literacy tend to have worse health outcomes, especially with regard to control of chronic conditions such as asthma and diabetes [7-9]. While the association between parental literacy and children’s health outcomes has been explored in depth in the medical literature, these relationships have only recently been examined in the context of dental health [10-12].

The majority of studies exploring the connection between parental literacy and their children’s health rely on self-reported behaviors and outcomes. In both research and clinical practice, adherence to preventive regimens is commonly assessed by self-report. However, the integrity of such assessments is contingent on the accuracy of subjects’ responses as well as the fidelity with which they report actual behaviors [13]. The validity of self-report measures may be influenced by a variety of factors, including parental recall ability, assessment mode, and social desirability bias (SDB).

### Social desirability bias

SDB is a form of response bias in which individuals misrepresent self-reported behaviors by over-reporting behaviors considered socially desirable, and underreporting undesirable ones [14]. SDB has been most thoroughly investigated in the social sciences, where it is considered one of the most significant and common threats to validity in behavioral sciences research [15]. Unfortunately, the methods utilized to control SDB are highly complex, and no currently available methods are capable of successfully eliminating this source of bias [16].

Although oral health behaviors are hypothesized to be a part of the mechanisms by which literacy can impact oral health status, our previous work found only a weak correlation between caregiver oral health literacy and oral health behaviors [11]. This result raised the question of potential biases, including the possible effects of social desirability bias (SDB), as our analysis used parental self-reported behavioral data. With this backdrop, the specific aims of the current investigation were: (1) to compare caregivers’ responses to traditional *versus* new oral health behavior (OHB) items designed to modulate SDB; and (2) to examine the association of caregiver oral health literacy (OHL) with reported OHBs.

## Methods

### Study sample

This was an IRB-approved cross-sectional study of 102 caregiver-child dyads presenting to a University-based dental clinic and a community-based general health clinic site. A sequential convenience sample of caregiver/child dyads was recruited. Both sites were participants in the Carolina Oral Health Literacy (COHL) Project [10]. Inclusion criteria included healthy children aged 6 years or younger with English-speaking primary caregivers. Children in this age group require more assistance with oral health behaviors, thus their oral health outcomes are more tightly linked to caregiver behaviors. The health literacy instrument utilized has been validated only in English, so only English-speaking caregivers were eligible for the study. Caregiver self-reported OHBs were collected using both traditional and new items designed to modulate SDB.

### Data collection procedure

Our data collection procedures have been described in detail in a previous publication [11]. To summarize, after obtaining written informed consent for study participation, eligible caregivers were asked to complete in-person, verbally-administered surveys by two trained interviewers in a private area. If the primary caregiver experienced any difficulty reading the consent, the interviewer read them aloud. The surveys were verbally administered by two trained interviewers who relied upon a standardized order of data collection as follows: the survey of the caregiver’s oral health knowledge, the survey of the child’s oral health behavior, the survey of the caregiver’s perception of oral health status and the caregiver’s literacy instrument. Reliance on this sequence prevented confounding of oral health knowledge, based on the behavior questions. Because the assessment of health literacy can be sensitive for some individuals, the oral health literacy instrument (REALD-30) was administered last.

### Variable measurement

OHBs were caregiver-reported and were assessed in the domains of child oral hygiene and feeding practices. To investigate our SDB hypothesis, we examined three pairs of behavior questions and compared caregivers’ responses to traditional and new SDB-modulating items in the domains of bottle-feeding, daily tooth brushing, and use of fluoridated toothpaste. The three ‘old’ behavior questions were time-honored items often found in dental history forms. Although not formally validated, they were introduced in the research arena by Douglass et al. and have been used by others [11,12,17-19]. These traditional questions, which we considered to be more SDB-vulnerable, were paired with reformulated items designed to reduce the impact of SDB. The traditional items asked “Do you clean or brush your child’s gums/teeth every day? Responses included 1) yes or 2) no. The new item asked “How often did you help your child brush their gums/teeth? Responses included 1) does not need help, 2) at least 2 times a day, 3) once a day, 3) less than once a day, and 4) once a week. We refer to these new questions as SDB-modulating items. The SDB-modulating items were more indirect. For bottle-feeding, we developed a SDB-modulating item by slightly rephrasing the traditional item. For the use of fluoridated toothpaste, the SDB-modulating item consisted of a multiple choice list that included other means of oral hygiene, as well as non-fluoridated toothpaste. We used identical items for daily brushing along with a new SDB-modulating item. Both the traditional and new SDB modulating items were asked within the same interview.

We measured caregivers’ oral health literacy using REALD-30, a validated word recognition test [20]. This instrument is comprised of 30 words used in dentistry, arranged in order of increasing difficulty. Administration of the REALD-30 requires the caregiver to read the words aloud to the interviewer. Caregivers are asked not to try to pronounce words they do not know, but simply to skip words that are not familiar. To score the test, one point is assigned for each word pronounced correctly, and the points are summed to give a total score. The total score can range from 0 (lowest literacy) to 30 (highest literacy).

Demographic data included caregiver race, age, education, number of children, marital status, household income, and study site. Race was self-classified as white, African American, Latina, or Asian. Age was categorized as a quartile-categorical variable. Education was coded as a four-level categorical variable where 1: did not finish high school, 2: high school completion or General Education Diploma (GED), 3: some technical or college education, and 4: college degree or higher. For descriptive purposes, marital status was coded as married, separated/divorced, or single/never married and as a dichotomous (married vs. single/never married/separated/divorced) for analytical purposes. Household income was grouped in three categories and was coded as 1: ≤$10,000, 2: $10,001-29,999, and 3: ≥$30,000.

### Analytical approach

Descriptive and summary estimates [simple proportions, mean, range, and standard deviation (SD)] were used for initial data presentation. The normality assumption of OHL scores was tested using a combined kurtosis and skewness X^2^ test [21]. To investigate the association of OHL with socio-demographic characteristics and OHBs, we first used analysis of variance (ANOVA) and a *p* < 0.05 criterion to evaluate OHL differences at the bivariate level. To quantify the association between OHL and parental report of “not brushing child’s teeth because it would be frustrating” we employed multivariate analysis based on log-binomial modeling. The selection of a log-binomial over a logistic model was based on the >20% frequency (24%) of the modeled outcome. Selection of covariates was based on bivariate testing results and evidence from the literature, whereas in the final model this was determined by a 10% change-in-estimate criterion of the OHL coefficient. We chose the change-in-estimate vs. a statistical significance criterion for the final model-building, because the former method has been shown to perform better under most conditions for control of confounding [22].

To illustrate the extent of agreement and the direction of discordance, we cross-tabulated responses to the above pairs of questions. For ease of interpretation, categories were collapsed and missing observations discarded to create 2 × 2 tables, as illustrated in the Additional file 1. We quantified the agreement between responses by calculating percent agreement (PA) and Cohen’s *kappa* statistic and 95% confidence limits (CL) obtained with bootstrapping (10,000 repetitions). We considered *kappa* in addition to PA because it quantifies the agreement beyond what would be expected by chance alone [23,24]. All analyses were conducted with Stata 11.2 (StataCorp LP, College Station, TX).

## Results

Our analytical sample consisted of 102 caregivers with mean age of 31 years (median 30 years). Socio-demographic characteristics and stratified OHL scores are presented in Table 1. Half of the caregivers were African American, half were married, and two-thirds had annual household income of less than $30,000. The mean REALD-30 score was non-normally distributed (X^2^ = 11.3; df = 2; p < 0.05) with mean 20.7 (SD = 7.0) and median of 22. We observed marked gradients in the OHL within levels of the examined covariates, particularly with regard to education (p < 0.0005) and income (p = 0.001). For example, the mean score was 14.7 among caregivers who had not finished high school vs. 18.0 among those with high school diploma or GED and 25.2 among those with college or higher education. Similarly, caregivers with household income of more than $30,000 had 5.9 points higher REALD-30 score compared to those with less than $10,000. We also noted marked racial differences with non-whites having a substantially lower mean REALD-30 when compared to whites [mean = 24.5 (SD = 4.6)]: African Americans—17.6 (SD = 7.8); others—21.6 (SD = 4.6); ANOVA p < 0.0005]. The number of children was the only covariate not showing a strong relationship with OHL scores.

**Table 1:** Distribution of Oral Health Literacy (OHL) scores by demographic characteristics among the participating caregiver-child dyads (n = 102)

	n^*^	**%**	**OHL (REALD-30)**		**ANOVA F value; (df1, df2), p**
			**Mean (SD)**	**Median (range)**	
**Race**					12.2; (2, 99); p < 0.0005^†^
White	36	35	24.5 (4.6)	26 (14-30)	
African American	49	48	17.6 (7.8)	18 (1-29)	
Other	17	17	21.6 (4.6)	21 (13-30)	
**Education**					13.0; (3, 98); p < 0.0005^†^
Did not finish high school	17	17	14.7 (8.7)	14 (1-30)	
High school diploma of GED	27	26	18.0 (6.9)	20 (2-29)	
Some technical or college	36	35	22.8 (4.8)	24 (13-30)	
College degree or higher	22	21	25.2 (3.8)	25.5 (28-30)	
**Number of children**					0.5; (3, 94); p = 0.7
1	28	29	19.6 (6.0)	20.5 (2-29)	
2	40	41	20.1 (7.7)	22 (1-30)	
3	20	20	21.2 (6.7)	22 (5-30)	
4 or more	10	10	22.4 (7.7)	25 (2-28)	
**Marital status**					6.7; (2, 99); p = 0.002^†^
Married	49	48	23.2 (5.2)	24 (13-30)	
Separated/Divorced	6	6	20.3 (9.1)	24 (2-26)	
Never married or single	47	46	18.2 (7.6)	21 (1-30)	
**Household income**					7.4; (2, 98); p = 0.001^†^
≤$10,000	40	40	18.1 (7.5)	19.5 (1-29)	
$10,001-30,000	27	27	20.0 (6.3)	22 (2-30)	
>$30,000	34	34	24.0 (5.6)	26 (4-30)	
**Site**					5.3; (1, 100); p = 0.02^†^
WIC clinic	55	54	19.3 (7.4)	22 (1-29)	
Dental School clinic	47	46	22.4 (6.3)	24 (4-30)	
**Age quartiles** (years)		Mean(SD)			3.6; (3, 95); p = 0.02^†^
Q1 (range: 18.2-25.5)	25	22.6(2.0)	17.7 (5.8)	18 (5-27)	
Q2 (range: 25.7-30.0)	25	27.5(1.2)	20.0 (7.6)	21 (1-29)	
Q3 (range: 30.3-34.6)	25	32.6(1.3)	22.0 (6.2)	24 (4-30)	
Q4 (range: 35.3-63.9)	24	42.2(6.8)	23.5 (6.6)	25 (2-30)	

*column totals may not add to total due to missing data; ^†^denotes a statistically significant association.

The contrast of the three pairs of old and new questions is presented in Table 2. Agreement was high for history of bottle-feeding [PA = 95%; kappa = 0.83 (95% CL = 0.68, 0.99)] and use of fluoridated toothpaste [PA = 88%; kappa = 0.67 (95% CL = 0.49, 0.85)] but was lower for daily tooth brushing [PA = 78%; kappa = 0.25 (95% CL = 0.04, 0.46)].

**Table 2:** Contrast and agreement [percent agreement (PA), and Cohen’s *kappa *and 95% confidence limits (CL^*^)] of caregivers’ responses between the three ‘old’ and ‘new’ Social Desirability Bias (SDB)-modulating items pertaining to child oral health-related behaviors

	**Old item: Do you clean or brush your child’s gums/teeth every day?**			**Old item: Do you use toothpaste when brushing your child’s teeth?**			**Old item: Was your child fed with a bottle?**	
**New item**^**†**^**: How often did you help your child brush their gums/teeth?**	**Yes**	**No**	**New item: Used toothpaste with fluoride to clean child’s teeth**	**Yes**	**No**	**New item: How often was your child fed with a bottle?**	**Yes**	**No**
Does not need help	7	0	Yes	66	6	More than 1 night/week	81	4
At least 2 times/day	45	1	No	5	16	Never	0	11
Once a day	20	2						
Less than once a day	12	6						
Once a week	5	0						
	PA = 78%			PA = 88%			PA = 95%	
	Kappa (95% CL) = 0.25 (0.04-0.46)			Kappa (95% CL) = 0.67 (0.49-0.85)			Kappa (95% CL) = 0.83 (0.68-0.99)	

*obtained with bootstrapping (n = 10,000 repetitions); †to enable the calculation of PA and *kappa* from a 2x2 table the question was converted to a binary item where: “at least 2 times/day” and “once a day” corresponded to “yes” and “does not need help”, “less than once a day” and “once a week” corresponded to “no”.

The new OHB items were used to assess the relationships between OHL and OHBs. Analysis of the association between OHL and behavioral covariates is presented in Table 3. Strong correlations were observed between REALD-30 scores and use of bottle feeding, as well with bedtime bottle or sippy cup usage. Parents with lower REALD-30 scores were more likely to report being frustrated “every time” or “never” when trying to clean their child’s teeth, and were more likely to report forgoing oral hygiene efforts due to frustration.

**Table 3:** Distribution of Oral Health Literacy (OHL; REALD-30) scores by child oral health-related behaviors among the participating caregiver-child dyads (n = 102)

	n^*^	**%**	**OHL (REALD-30)**		**ANOVA**
			**Mean (SD)**	**Median (range)**	**F value; (df1, df2), p**
**Did you ever get frustrated trying to clean your child’s teeth?**					3.2; (3, 98); p = 0.03^†^
Every time	11	11	16.5(7.4)	18(4-27)	
Sometimes	32	31	22.8(5.9)	23.5(10-30)	
Hardly ever	9	9	23.6(4.3)	25(13-28)	
Never	50	49	19.8(7.5)	22(1-30)	
**Did you ever decide not to clean his/her teeth because it would be frustrating?**					4.7; (1, 100); p = 0.03^†^
Yes	24	24	18.0(7.6)	18(2-30)	
No	78	76	21.5(6.7)	23(1-30)	
^**‡**^**How often did you help your child clean/brush their gums/teeth?**					0.5; (3, 98); p = 0.7
At least 2 times/day	48	47	19.9(7.7)	21.5(2-30)	
Once a day	23	23	21.0(6.5)	23(1-28)	
Less than once a day	23	23	21.0(6.5)	23(1-28)	
Does not need help	8	8	23.0(5.1)	23(15-30)	
**If your child brushed their own teeth by themselves, how often did they brush?**					0.6; (3, 98); p = 0.6
At least 2 times/day	33	32	21.1(6.8)	22(4-30)	
Once a day	20	20	20.4(7.9)	21.5(2-30)	
Less than once a day	15	15	22.5(4.8)	22(14-30)	
Does not brush on his/her own	34	33	19.7(7.6)	22.5(1-29)	
**How many times a week did your child go to bed with a bottle or sippy cup?**					3.1; (3, 97); p = 0.03^†^
Every night	13	13	20.9(5.1)	21(24-29)	
Less than every night	11	11	21.2(6.4)	23(7-30)	
Used to, but stopped	26	25	17.3(8.2)	19.5(1-29)	
Never goes to bed with a bottle	51	50	22.3(6.5)	24(4-30)	
**How often did your child use a sippy cup?**					0.7; (2, 99); p = 0.5
At least 2 times/day	43	42	21.6(5.6)	22(7-30)	
Once a day or less frequently	9	9	20.9(7.1)	23(12-30)	
Does not use a sippy cup	50	49	19.9(8.1)	22(1-30)	
^**‡**^**Was your child fed with a bottle?**					8.7; (1, 99); p = 0.004^†^
Yes	89	87	20.0(7.1)	22(1-30)	
No	12	12	26.2(3.5)	26.5(20-30)	
**How many months was your child fed using a bottle?**					
0-6 months	16	17	21.9(7.3)	25.5(4-30)	2.1; (3, 91); p = 0.1^†^
7-12 months	47	49	18.7(7.7)	21(1-30)	
13-18 months	16	17	23.3(3.3)	24(17-29)	
19+ months	16	17	20.6(6.4)	22.5(4-30)	
**What did you use to clean your child’s teeth?**					n/a^¶^
Toothbrush only	60	59	19.8(7.1)	21(1-30)	
^**‡**^Fluoridated toothpaste	76	75	20.7(7.5)	23(1-30)	

*column totals may not add to total due to missing data; † denotes a statistically significant association; ^**‡**^denotes items that were used in the SDB analysis; ¶ no formal statistical analysis was conducted for this item which allowed for multiple possible responses.

Table 4 presents results of the multivariate analysis quantifying the association between OHL and the prevalence of caregiver report of “decided not to brush the child’s teeth because it would be frustrating”. Among the variables that were evaluated as confounders in models B and C (age, race, education, income, number of children, study site) only race, marital status and study site met the 10% change-in-estimate criterion for inclusion in the final model (Model D). Using this final model (D) it was evident that while controlling for caregivers’ race and study site, one-unit increase in OHL corresponded to a 6% decrease in caregiver report about frustration (adjusted PR = 0.94; 95% CL = 0.89, 0.98).

**Table 4:** Multivariate log binomial regression modeling results of caregiver report “Decided not to brush my child’s teeth because it would be frustrating” [(adjusted prevalence ratio (PR) and 95% confidence limits^*^ (CL)] on Oral Health Literacy (OHL) among the WIC/UNC participants (n = 102)

	**Model A**	**Model B**	**Model C**	**Model D**^**‡**^
	**PR (95% CL)**	**PR (95% CL)**	**PR (95% CL)**	**PR (95% CL)**
**Oral health literacy** (REALD-30 score)^†^	0.96 (0.92, 0.99)	0.96 (0.92, 0.99)	0.95 (0.92, 0.98)	0.94 (0.89, 0.98)
**Age** (years; quartiles)		1.24 (0.88, 1.74)		
**Site** (*referent*: University clinic)				
WIC site			2.68 (1.31, 5.50)	2.55 (1.18, 5.53)
**Race***(referent: Whites)*				
African American				1.48 (0.43, 5.15)
Other				0.97 (0.37, 2.53)
**Marital status** (*referent: married*)				
Single/never married/separated/divorced				0.42 (0.13, 1.29)
Change in estimate (percent)	ref	vs. A: 6.4	vs. A: 16.5	vs. C: 14.7

*confidence limits were based on robust standard errors; †estimate corresponds to one-unit change in REALD-30 score; ‡ denotes the ‘final’ Model; the education, income, and number of kids variables did not meet the 10% change-in-estimate criterion and thus were not included in the final model (Model D).

## Discussion

The major aims of this investigation were to 1) examine SDB in caregiver reports of preventive oral health behaviors, and 2) examine the association between OHL and oral health behaviors. We found evidence of SDB in 2 of 3 examined behaviors, with the greatest discordance in the item regarding toothbrushing frequency. Using indirect questioning methods appears to mitigate SDB and provide a better estimate of oral health behaviors. Dental caries is a common chronic disease of childhood, and its prevention for young children hinges upon caregiver assistance with OHBs. For this reason, accurate assessment of caregiver-reported OHBs is essential in developing interventions to reduce dental disease in children.

Although the influence of SDB on self-reported health behaviors has long been recognized, research on the impact of SDB in medicine and dentistry has been limited by the methodological difficulties of detecting, quantifying, and controlling this form of bias. To date, much of the published literature has focused on the effects of SDB on dietary self-reports, or on highly sensitive behaviors such as smoking, sexual conduct, and illicit drug usage [25-29]. In these settings, the tendency of individuals to answer in socially desirable ways, as measured by social desirability scales, has been positively associated with self-reported preventive health behaviors in both adults and children [27]. Sjöstrom and Holst investigated the impact of SDB on response error in a dental questionnaire, comparing self-reported levels of preventive dental visits to insurance claim data in a Swedish population. Differences between reported and actual dental attendance ranged from 4.4-42.7%. Perhaps more importantly, individuals overstated their reported number of dental visits by a greater amount when their actual level of attendance deviated more significantly from the social norm [30]. Thus, individuals appeared to be fully aware of the norm, modifying their responses in such a way as to minimize reporting of socially undesirable behaviors.

Previous investigations relied on individual self-reports to examine SDB. Our study is unique in that we examined SDB in caregiver-reported behaviors. It has been noted that multi-item, indirect responses may modulate social desirability bias compared to binary response items. Our study was the first to develop such multi item indirect responses. In pediatric practice, we often depend on caregiver-reported behaviors to provide accurate risk assessment; accordingly, understanding the effects of SDB on caregiver self-reports is an important aspect of providing timely and appropriate anticipatory guidance.

Our results confirmed the association between lower literacy and deleterious OHBs, as previously reported [11,12]. This was evident in 4 of 8 OHBs examined relating to oral hygiene and feeding practices. Although a formal statistical analysis was not conducted for a 9th item that allowed multiple responses with regard to means of cleaning a child’s teeth, we observed that caregivers who responded ‘fluoridated toothpaste’ had on average one point higher REALD-30 score compared to those who responded “toothbrush only”. We were struck by the strength of the association between literacy and avoidance of tooth brushing due to frustration. Anecdotally, some of the frustration experienced by parents appears to stem from a lack of information from health care providers regarding practical aspects of performing oral hygiene behaviors. Today, parents who perceive a lack of knowledge often turn to the internet or other social media sources [31,32], where a multitude of parenting blogs and web boards provide such shared information. Frustration appears to be a barrier preventing some parents from consistently performing oral hygiene tasks. As current pediatric dental interventions typically fail to address the psychological aspects of parental hygiene assistance, this represents an opportunity to strengthen caregiver oral health education and anticipatory guidance.

These results should be interpreted in consideration of the study’s limitations. Word recognition tests, such as the REALD-30 and the REALM, assess reading ability and have been shown to be reliable proxies of literacy in English speakers [20,33-35]. However, these instruments are not capable of assessing the wide array of skills that comprise health literacy, including health knowledge, numeracy, and receipt of information via other modes of communication. Because REALD-30 has been validated only in English, we were unable to recruit non-English speaking respondents.

Given these limitations, we believe there were several strengths in our study. One is the use of trained examiners to administer in-person interviews for data collection. Although the effects of SDB on self-report measures may never be completely eliminated, there is some evidence that face-to-face interviews yield more accurate responses than self-administered computer interviews or paper questionnaires [13]. This interview mode also eliminates the need for participants to read the questionnaire, diminishing the potential for inaccurate responses secondary to insufficient reading skills. Additionally, since low literacy can be a source of sensitivity or shame for individuals, the REALD-30 is administered at the end of the interview process to minimize the potential for bias induced by psychological distress related to literacy measurement.

## Conclusion

Under the conditions of this study, we found evidence of SDB in caregiver-reported OHBs. Use of SDB-modulating items to elicit information regarding dental behaviors may yield more accurate information in some dimensions, such as toothbrushing. However, information on the effects of SDB on oral health self-reports remains incomplete. Future studies should explore the impact of SDB on self-reported preventive behaviors, and how survey items may be designed to minimize this form of bias.

## Ethics committee

This study was approved by the UNC Biomedical Institutional Review Board.

## Abbreviations

OHBs: Oral health behaviors; OHL: Oral health literacy; OHS: Oral health status; REALD-30: Rapid estimate of adult literacy in dentistry-30; SDB: Social desirability bias.

## Competing interests

The authors declare that they have no competing interests.

## Authors’ contributions

LS was the lead author and coordinated the design, data collection and writing of the investigation. JL is LS is mentor and principal investigator of the overall research project. She was involved with every aspect of the project from implementation, design, execution and writing of the manuscript. KD conducted the data analysis, assisted with interpretation of the results and writing of the manuscript. DD assisted in interpretation of the data and writing of the manuscript. DB coordinated and conducted data collection. WV assisted in interpretation of the data and writing of the manuscript. All authors read and approved the final manuscript.

## Pre-publication history

The pre-publication history for this paper can be accessed here:

http://www.biomedcentral.com/1472-6831/13/24/prepub

## Supplementary Material

Additional file 1 Model of 2x2 table used to illustrate the extent of agreement and the direction of discordance between old, traditional OHB items and new, SDB-modulating items. Click here for file
