# Supplementary material for: A cross sectional study examining social desirability bias in caregiver reporting of children’s oral health behaviors
Source: BMC Oral Health. 2013 Jun 1;13:24. doi: 10.1186/1472-6831-13-24 (PMC3680187; doi:10.1186/1472-6831-13-24)
Supplement: Additional file 1 — Model of 2x2 table used to illustrate the extent of agreement and the direction of discordance between old, traditional OHB items and new, SDB-modulating items. [file 1472-6831-13-24-S1.doc]

**Supplemental Table Model of 2x2 table used to illustrate the extent of agreement and the direction of discordance between old, traditional OHB items and new, SDB-modulating items.**

|  |  | OLD ITEM | |  |
| --- | --- | --- | --- | --- |
|  |  | Yes | No |  |
| NEW ‘SDB’ ITEM | Yes |  | B |  |
|  | No | A |  |  |
|  |  |  |  |  |
| SDB Hypotheses:  H_a_: Pr(Old=yes & New=no) > Pr(Old=no & New=yes)  H_0_: Pr(Old=yes & New=no) ≤ Pr(Old=no & New=yes) | | | | |

SDB Hypothesis:

H_a_: Pr(Old=yes & New=no) > Pr(Old=no & New=yes)

H_0_: Pr(Old=yes & New=no) ≤ Pr(Old=no & New=yes)
